# Supplementary material for: Natural resources modulate the nexus between environmental shocks and human mobility
Source: Nat Commun. 2023 Mar 13;14:1393. doi: 10.1038/s41467-023-37074-y (PMC10011366; doi:10.1038/s41467-023-37074-y)
Supplement: Supplementary file 1 — Supplementary Information [file 41467_2023_37074_MOESM1_ESM.pdf]

Supplementary Information for *Natural Resources Modulate the Nexus Between Environmental Shocks and Human Mobility*

Michael Brottrager[a], Jesus Crespo Cuaresma[b,c,d], Dominic Kniveton[e,f], and Saleem H. Ali[f,g,2]

[a]Department of Economics, Johannes Kepler University, Linz, Austria; [b]Department of Economics, Vienna University of Economics and Business, Vienna, Austria; [c]Population and Just Societies Program, International Institute for Applied Systems Analysis, Laxenburg, Austria; [d]Wittgenstein Centre for Demography and Global Human Capital, Vienna, Austria; [e]School of Global Studies, University of Sussex, Brighton, UK; [f]United Nations International Resource Panel, Paris, France; [g]Department of Geography and Spatial Sciences, University of Delaware, Newark, US

To estimate the effects of drought on migration decisions, we make use of both aggregated international data as well as spatially disaggregated (grid-level) data. Our two main sources of population and migration data, respectively, are migrant stock dataset (1960-2000) from (1) and the Gridded Population of the World (GPW) dataset.

For estimates based on internal migration, we employ the Gridded Population of the World (GPW), under the assumption of stability in birth and mortality rates around a global trend at the cell level over the period under study. The data existing at the required level of scope and disaggregation do not allow for a better measurement of human mobility, although current efforts aimed at estimating migration at a high spatial resolution may provide more precise information in the near future (2).

To construct the SPEI, we make use of climate information provided by the Climatic Research Unit (CRU) (3) and we evaluate the value of the index around location specific growing seasons for the most important crop. To do so, we use FAO crop data on production and harvest areas and crop calendars extracted from a global data set of irrigated and rainfed crop areas around the year 2000 (4). Using location-specific growing season SPEI evaluations allows us to directly link SPEI and its behavior over the growing cycle to capture the role environmental shocks play as a determinant of agricultural productivity.

For our international migration analysis, we compare SPEI trajectories across countries and decades and employ differences in period-average SPEI scores compared to long term levels. Those periods range from a length of two to ten years and are then compared to 20 year country averages using the following equation

$$SP_{itp} = \frac{p^{-1}\sum_t^p SPEI_{it}}{MSPEI_{it}^{20}}, \text{ for } p = 1, \ldots, 10$$

with $MSPEI_{it}^{20} = 20^{-1}\sum_t^p SPEI_{it}$. Lower values of SP_{itp} are associated with falling profiles in SPEI under the condition that the overall trajectory is close to linear.[*] In that sense, estimation results using the SPEI trajectory measure are to be interpreted as long run (improving) worsening climate conditions as opposed to single events of droughts and thus capture (decreased) increased drought probability over the observational period.

Worsening SPEI scores are indicated by lower period-averages compared to the country-specific long term levels. Comparing effect size estimates using such a variable allows us to shed light on long-run migration responses to worsening climate conditions and by extension can be related to worsening land-based natural resources. Following (5), at the grid level we construct a measure of drought periods as the proportion of months with SPEI scores below -1.5 out of the past 12 months. When the longest streak starts in the previous year, it is counted and included in the year in which the streak ended, so theoretically the proportion can be above unity. We aggregate this measure for the past two years to capture longer drought periods, as grid-level migration data is only available every five years. Land degradation data in the form of changes in soil carbon stocks comes from the Trends.earth project (6). The use of soil carbon stock degradation as a variable instead of land productivity or land use change is based on the fact that the latter are very likely to be mechanically impacted by population density, which is our output variable. Changes in carbon stock are slow moving and, by construction, less likely to suffer directly from this reverse causality bias. Carbon stock is the quantity of carbon in a reservoir which has the capacity to accumulate or release carbon and comprised of above and below-ground biomass, dead organic matter, and soil organic carbon. The original source is the Soilgrids.org database (7). It provide soil organic carbon content for the entire globe at 250 metres resolution and for 7 different soil depth (0 cm, 5 cm, 15 cm, 30 cm, 60 cm, 100 cm and 200 cm). The trend.earth project approximates soil carbon stock degradation between periods by multiplying changes in soil organic carbon by a set of conversion coefficients. Areas experiencing a decrease of at least 10% of their soil organic carbon stocks are considered degraded. Our degradation variable takes the share of degraded area within each cell.

Figure 1 shows the estimates of the effects of a decrease in drought risk (that is, an increase in the SPEI variable) on international emigration rates, as measured by the dataset in (1). The effects are presented for different subsamples of countries defined by income per capita (in four groups), and for different duration lengths of the change in SPEI. Lower values of the variable are associated with falling profiles in SPEI (assuming that the overall trajectory is close to linear) and lead to decreases in emigration for the poorest countries in our sample, in particular for long-lasting trends.

Existing quantitative research linking the environment and migration has tended to focus on the marginal influence of temperature and rainfall on migration flows. One of the key

[*]Clearly, one limitation of this approach is its weakness in capturing highly non-linear SPEI trajectories, that is (inverse-)U-shaped SPEI trajectories. Given that it is average period SPEI and not yearly SPEI realizations that enter the equation above, this issue should not affect the analysis too much.

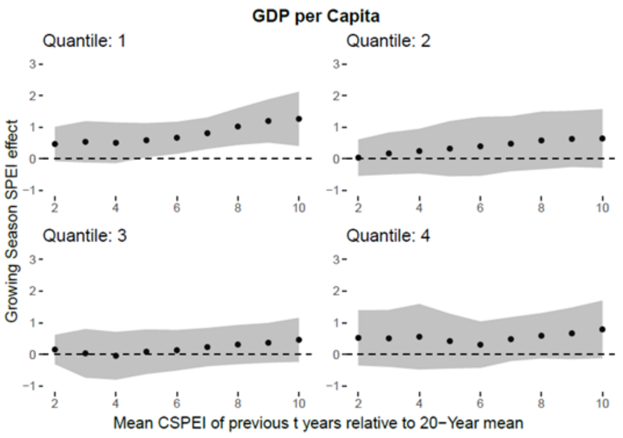

Fig. 1. Growing-season SPEI (CSPEI) trajectory effect estimates on log international emigration rates. The mean effect estimates with 95% confidence intervals are presented for different bandwidths along the horizontal axis, proxying short (2) to long lasting (10) periods of worsening drought conditions. Standard errors are clustered at the regional level. The model also includes region-by-decade fixed effects to account for potential regional confounders, n=111

findings in recent years in the context of global climate change has been the contrasting impact of higher temperatures on international migration from middle-income economies compared to that from poorer countries. In the former, increased temperatures are associated with increased migration rates to other countries. In poorer countries, the reverse is observed, with higher temperatures decreasing the probability of migration to other countries (8). The reasons for this difference have been suggested to be that international migration is a costly process for those involved and in poorer countries, with predominantly agriculturally based livelihoods, high temperatures are associated with a reduced probability of would-be migrants having the resources to move.

While much of the past research in this area has focused on the linkages between climate and migration, less attention has been on the role of natural resources contributing to decreases or increases in migration. This is despite the causal pathway linking climate to migration often being assumed to occur via the influence of climate shocks and stresses on natural resource-based livelihoods, such as agriculture. In part, the neglect of looking for a natural resource-migration link is related to the potential sensitivity of the resource variable to increased or decreased populations from migration as well as being a potential cause of migration. For example, improved land productivity could be a cause of migration, through being more able to afford to migrate, as well as a result of decreased population pressure on land due to outmigration. With climate related variables, this reverse causality is not present, as migrant flows are generally accepted to be extremely unlikely to cause changes in the climate. Reverse causality is a problem when trying to identify the marginal influence of natural resources on migration. In recognition of this, the approach taken is to look at a common stressor on natural resources, namely drought, and its statistical association with migration. Drought is widely recognized to have a negative impact on agricultural productivity. It should be born in mind that other non-climate stressors can also influence agricultural productivity, but here we focus on the particular role played by drought. In a number of countries, agricultural productivity is highly linked to the strength of the economy, implying that drought can also affect non-agricultural sectors, albeit to lesser degree (9). The agricultural sector is still a highly relevant source of income in many developing countries, in particular in rural areas.

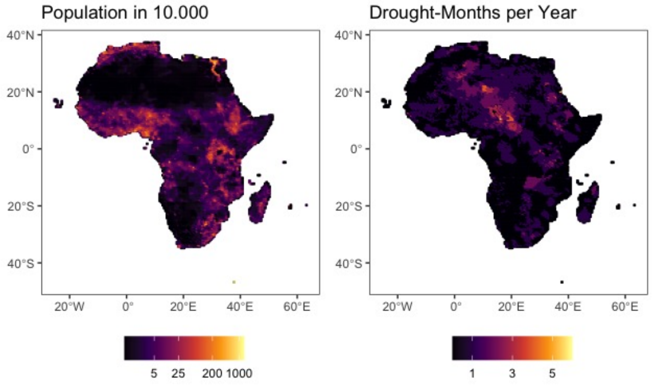

Fig. 2. Population (logged) and drought duration (drought months per year, identified by growing season SPEI lower than -1.5) by cell evaluated in the year 2000.

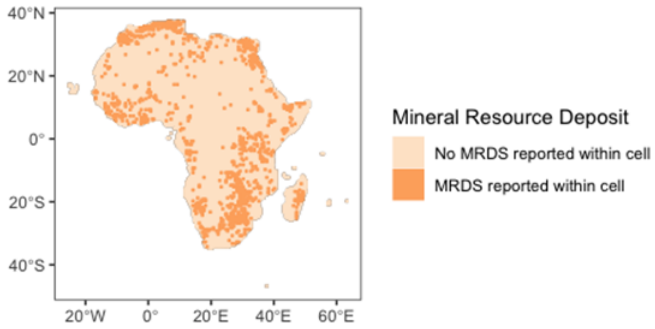

Fig. 3. Currently active and past locations of mineral resources extraction sites (MRDS). MRDS are aggregated within each cell.

Figure 2 displays cells that were subject to a drought in the year 2000, together with population estimates. As can clearly be seen, drought periods are clustered, raising the issue of potential spatial autocorrelation which would need to be accounted for in our estimation model. Furthermore, severe drought events affect cells with very low population densities.

Figure 3 displays both active and currently inactive mineral resource deposits. As we do not have data on actual extraction volumes at hand, resource deposits aggregated to the grid level are used to proxy for potential sources of income different from agriculture.

Table 1 presents the regression results of a model at the grid-cell level including both interaction terms with the resource variable and income quartile dummies. The results indicate that the difference in the strength of the effect is better explained by differences in income level, and that once we control for economic development, the interaction term with the resource covariate appear significant. The correlation between income and mineral resources is likely to be driving the insignificant effects found for the resource when we allow for different sources of parameter heterogeneity in the regression specification.

Table 1. Interaction Model

Drought event	0.068
	(0.056)
× Minerals in cell	0.038
	(0.065)
× 1st Income quartile	-0.441***
	(0.105)
× 2nd Income quartile	-0.288***
	(0.100)
× 3rd Income quartile	-0.057
	(0.052)
Cell FE	Yes
Year FE	Yes
Observations	37716
R^2	0.015

Standard errors in parentheses
Two-sided t-tests, * p<0.1, $^{*}*$ p<0.05, $^{*}**$ p<0.01

1. Ç Özden, CR Parsons, M Schiff, TL Walmsley, Where on earth is everybody? the evolution of global bilateral migration 1960–2000. *The World Bank Econ. Rev.* **25**, 12–56 (2011).
2. A Alessandrini, D Ghio, S Migali, *Estimating net migration at high spatial resolution; Publications Office of the European Union, Luxembourg.* (2020).
3. I Harris, TJ Osborn, P Jones, D Lister, Version 4 of the cru ts monthly high-resolution gridded multivariate climate dataset. *Sci. data* **7**, 1–18 (2020).
4. FT Portmann, S Siebert, P Döll, Mirca2000—global monthly irrigated and rainfed crop areas around the year 2000: A new high-resolution data set for agricultural and hydrological modeling. *Glob. Biogeochem. Cycles* **24** (2010).
5. J Laurent-Lucchetti, M Couttenier, T Vischel, X Vollenweider, *Droughts, Land Degradation and Migration. United Nations Convention to Combat Desertification (UNCCD).* (2019).
6. M Gonzalez-Roglich, et al., Synergizing global tools to monitor progress towards land degradation neutrality: Trends. earth and the world overview of conservation approaches and technologies sustainable land management database. *Environ. Sci. & Policy* **93**, 34–42 (2019).
7. T Hengl, et al., Soilgrids250m: Global gridded soil information based on machine learning. *PLoS one* **12**, e0169748 (2017).
8. C Cattaneo, G Peri, The migration response to increasing temperatures. *J. Dev. Econ.* **122**, 127–146 (2016).
9. M Burke, SM Hsiang, E Miguel, Global non-linear effect of temperature on economic production. *Nature* **527**, 235–239 (2015).
